# Muscle synergy space: learning model to create an optimal muscle synergy

**DOI:** 10.3389/fncom.2013.00136

**Published:** 2013-10-15

**Authors:** Fady Alnajjar, Tytus Wojtara, Hidenori Kimura, Shingo Shimoda

**Affiliations:** ^1^Intelligent Behavior Control Unit, Brain Science Institute, BSI-TOYOTA Collaboration Center of RIKENNagoya, Japan; ^2^Department of Mechanical Engineering, Division of Artificial Systems Science, Chiba UniversityChiba, Japan

**Keywords:** muscle synergy space, synergy stability index, synergy coordination index, automatic posture response

## Abstract

Muscle redundancy allows the central nervous system (CNS) to choose a suitable combination of muscles from a number of options. This flexibility in muscle combinations allows for efficient behaviors to be generated in daily life. The computational mechanism of choosing muscle combinations, however, remains a long-standing challenge. One effective method of choosing muscle combinations is to create a set containing the muscle combinations of only efficient behaviors, and then to choose combinations from that set. The notion of muscle synergy, which was introduced to divide muscle activations into a lower-dimensional synergy space and time-dependent variables, is a suitable tool relevant to the discussion of this issue. The synergy space defines the suitable combinations of muscles, and time-dependent variables vary in lower-dimensional space to control behaviors. In this study, we investigated the mechanism the CNS may use to define the appropriate region and size of the synergy space when performing skilled behavior. Two indices were introduced in this study, one is the *synergy stability index* (SSI) that indicates the region of the synergy space, the other is the *synergy coordination index* (SCI) that indicates the size of the synergy space. The results on automatic posture response experiments show that *SSI* and SCI are positively correlated with the balance skill of the participants, and they are tunable by behavior training. These results suggest that the CNS has the ability to create optimal sets of efficient behaviors by optimizing the size of the synergy space at the appropriate region through interacting with the environment.

## Introduction

Human behaviors are a result of complex neural dynamics between the central nervous system (CNS), proprioceptors, and muscles. Owing to redundancy in the human musculoskeletal system, the CNS can choose the most efficient movements from an infinite number of behavior options (Bernstein, 1967; Sporns and Edelman, 1993). Many researches have attempted to clarify the computational mechanism employed by the CNS for behavior selection by introducing the optimal parameters of behavior control (Flash and Hogan, 1985; Uno et al., 1989; Alexander, 1997), the principles of biological controllers (Tanaka and Kimura, 2008; Shimoda and Kimura, 2010; Shimoda et al., 2013) and other methods, but this problem remains an open research topic.

One possible mechanism for choosing a proper behavior from the numerous options is that through behavior training, the CNS narrows down the redundancy of the options to a smaller set. Choosing the behavior from a smaller set would allow the CNS to control the behavior with less redundancy. In such a scenario, the redundancy of the musculoskeletal system contributes toward creating a suitable set depending on the environment. In this paper, we discuss this scenario and demonstrate through experiments that the CNS can create an optimal set of efficient behaviors through behavior training.

**Figure 1:**
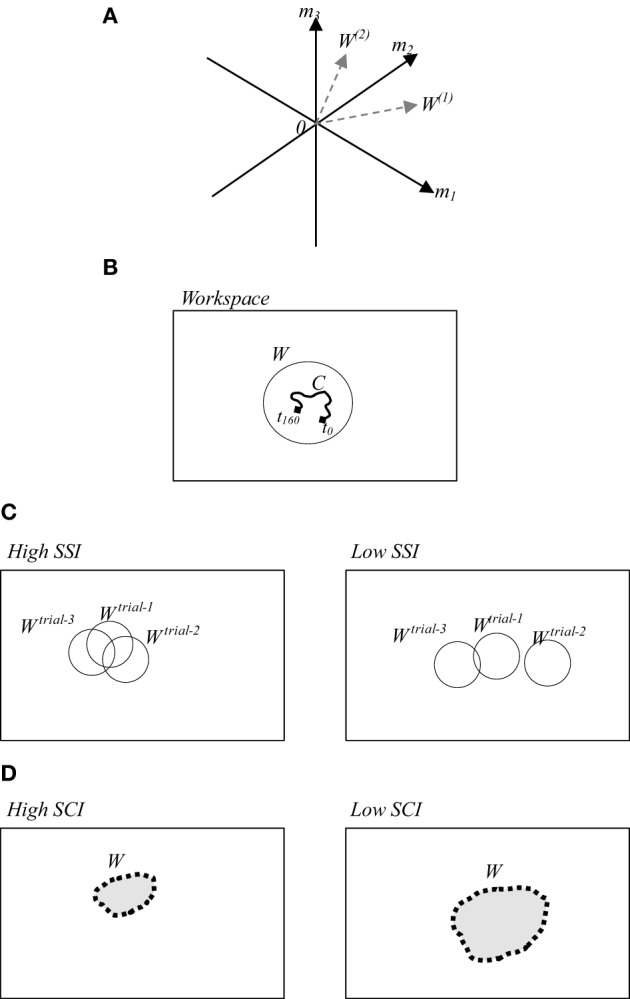
**(A)** A conceptual image of muscle synergy that illustrates two synergies *W*^(1)^ and *W*^(2)^ abstracted from three muscles activations *m*_1_, *m*_2_, and *m*_3_. **(B)** A simplified workspace where *W* represents an example of the selected solution from possible motions, and *C* represents the selected space in time of the selected solution. **(C)** (Left/right) examples of high/low SSI of three trials. **(D)** (Left/right) examples of utilized synergy in a trial with high/low SCI.

The notion of muscle synergy is introduced to simplify the computational mechanism between the CNS and muscle control (Popovic and Popovic, 2001; Cirstea et al., 2003; D'Avella et al., 2003; Krishnamoorthy et al., 2003; Sohn and Hallett, 2004; Tresch and Jarc, 2009; Clark et al., 2010; Safavynia et al., 2011; Cheung et al., 2012). Muscle synergy is defined by using several modules formulated by either time-dependent or time-independent parameters. In time-dependent formulations, muscle synergy constitutes the coordinated activations of groups of muscles with fixed time-varying profiles (D'Avella et al., 2006). In contrast, in time-independent formulations, muscles are activated in synchrony with fixed weights (Ting and Macpherson, 2005; Cappellini et al., 2006). These weights can create a new workspace whose dimensionality is lower than the number of muscles. A conceptual image of the workspace is illustrated in Figure 1A. Here three dimensional space of three muscle activations *m*_1_, *m*_2_, and *m*_3_ are reduced into the two dimensional synergy space represented by *W*^(1)^ and *W*^(2)^. Figure 1B illustrates a simplified version of the synergy space *W*. To recruit a behavior from this synergy space a time-dependent variable referred to as *neural command* (C) is used.

In this study, we adopted the time-independent synergy to reduce the number of behavior options by creating synergy space *W* corresponding to a suitable set containing only the efficient behaviors. Here the question to be addressed is how the CNS defines the appropriate region and size of the synergy space. To discuss on this issue, we computed the muscle synergy in the slightly different way from the conventional method (Torres-Oviedo and Ting, 2007). In the previous studies, mainly the muscle synergy is computed by averaging the data on several trials. However, we computed the synergy in each trial to emphasize the similarity of the synergies between the trials. Here we introduce two indices referred to as the *synergy stability index* (SSI) (Wojtara et al., 2012a) and the *synergy coordination index* (SCI). *SSI* indicates the similarity between synergy spaces of repeated similar behaviors. The higher value of SSI implies that the synergy space of the participant is fixed within a certain range as illustrated in Figure 1C (left). *SCI*, on the other hand, indicates the size of the synergy space. Higher *SCI* implies a smaller size of synergy space, Figure 1D (left). Detailed definitions of these two indices are provided in the following section.

To analyze behaviors using these two indices, we conducted experiments on automatic posture response (APR) in humans (Horak and Diener, 1994; Carpenter et al., 1999, 2001; Bloem et al., 2002). The importance of studying this task has been reported by many researchers due to its close relationship to the overall posture balance of the human body (Tsuruike et al., 2003; Torres-Oviedo and Ting, 2007; Wojtara et al., 2012b). Understanding the neural basis underlying posture balance control is a challenging research topic that can provide insight into properties of motor neural dynamics, as well as assistance in predicting and preventing the risk of falls and thus, their consequent harm (Tinetti et al., 1986; Bloem et al., 2003; Vassallo et al., 2005).

Through the APR experiments, we show in this paper the *SSI* and *SCI* are strongly related to the balance capability of the participants. Furthermore, these two indices are tunable by the behavior training. These results show that the CNS can optimize the size of the synergy space at the appropriate region through behavior training.

## Materials and methods

### Experimental setup and data collection

Two APR experiments were conducted in this study. The first experiment was an investigation of the relationship between the lateral balance ability of participants and the characteristics of their muscle synergies quantified by *SSI* and *SCI*. The second experiment was a training experiment to explore the plasticity of muscle synergy during training.

The participants in the first experiment were 8 healthy men (mean age 35.1 ± 8 years, mean weight 74 ± 18 kg, mean height 173.5 ± 12 cm). The participants in the second experiment were three out of the eight participants in the first experiment who showed the weakest ability to maintain balance. All participants were right-footed, and had no reported neurological disorders. The protocols of all experiments were approved by the RIKEN ethics committee.

**Figure 2:**
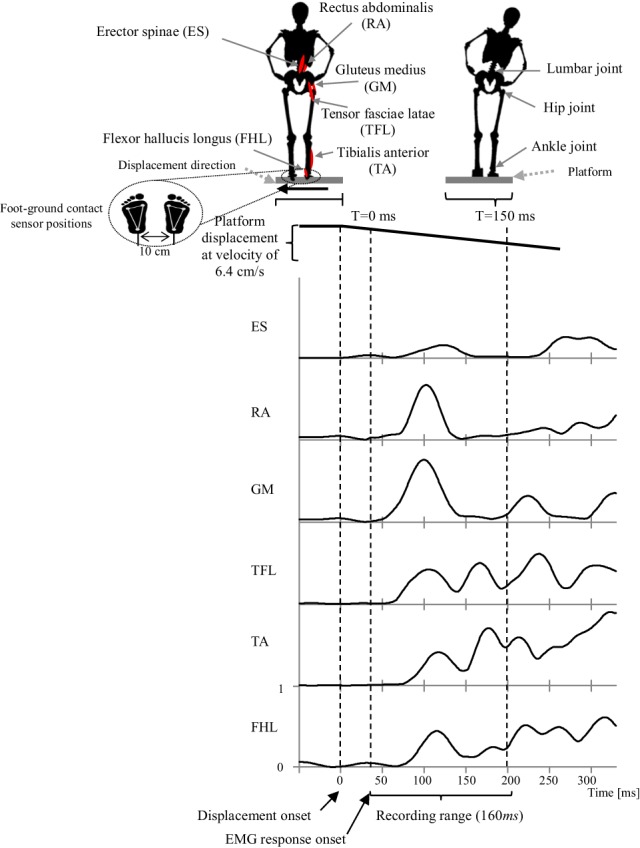
**Experimental setup that illustrates the experiment scenario, muscles locations, joint locations, platform motion and displacement speed, EMG recorded range and a representative EMG activities of the muscles in response to the platform displacement**. Subject EMG responses were in average occurring at latency of 40 *ms* after displacement onset. Thus, we consider this point as a starting time to record a 160 *ms* range of EMG data.

In the experiments, we instructed participants to stand upright in the *akimbo* position on a movable platform, placing their feet on foot-ground contact sensors located 10 cm apart (Figure 2). We then triggered a lateral displacement of 11 cm with velocity of approximately 6.4 cm/s using the platform. We instructed the participants to make an effort to maintain their balance when the platform was displaced (i.e., avoid body movements other than lateral hip flexion/extension and/or ankle inversion/eversion). The direction and timing of displacement were random and not predictable during the experiment. Before starting the experiment, we asked the participants to practice on the platform for 20 min (approximately 80 platform displacements with various velocities punctuated by some intermittent breaks) to warm up and get used to the experimental environment.

Each participant experienced approximately (mean ± SD: 18 ± 4) leftward and rightward platform displacements and only trials of the leftward displacements were used for analysis in order to avoid any mixing the differences in behavior arising from using the dominant foot as compared with the non-dominant foot (Torres-Oviedo and Ting, 2007).

Electromyography (EMG) wireless surface electrodes (BTC Free EMG, sampled at 1 kHz) were used to record data from six major leg and lower-back muscles of the subject's right side (Konrad, 2005). Muscles include; flexor hallucis longus (FHL), tibialis anterior (TA); which support the ankle strategy in lateral perturbation. In addition to the tensor fasciae latae (TFL), gluteus medius (GM), rectus abdominalis (RA), erector spinae (ES); which support the hip strategy in lateral perturbation (Runge et al., 1999, see Figure 2). EMG Electrodes were placed in accordance to the guidelines of the Surface Electromyography for the Non-Invasive Assessment of Muscles (SENIAM)–European Community project (Hermens et al., 1999).

Since we were interested in the initial stage of the APR, the first recorded 160 ms of muscle activities, after the EMG response onset, were used for synergy calculations. The entire time-series EMG data was rectified and processed with a low-pass filter with a cutoff frequency of 32 Hz. EMGs were normalized by their maximum mean measured during the experiment.

### Scoring participants' balance skill

A scoring system was applied to each participant to evaluate his balance skill in responding to the sudden platform displacement. Scores were displayed to the participant throughout the experiment on a front screen, in order to increase motivation to maintain balance. The scores were also used as a reference to associate balance skill with the resulting synergy characteristics of the participants. To estimate the scores, we visually observed each participant responses to each platform displacement and scored the response according to the criteria listed in Table 1.

**Table 1:** **Scoring system**.

**Case**	**Score**
Participant does not raise a hand or foot from the initial akimbo position	+2
Participant moves a hand from the initial akimbo position	+1
Participant lifts a foot from the initial position	−1
Participant totally loses his balance and moves a foot off the platform	−2

Although the scores were considered for all the trials, we computed muscle synergy using only the trials of similar quality responses of the subjects (score = +1), to avoid any mixing the differences in behavior arising from using different response strategies such as step strategy or falling. Experimentally, 5 trials were the common number of trials among the subjects that meet this conditions.

### Computing muscle synergies

An important feature of using muscle synergy is to reduce the dimensions of controlling the muscles. When *m* muscles are controlled, *m* commands are generally required.

Let us express the EMG data of *m* muscles by using the matrix *M*:

(1) $$M\in R^{m\;\times \;t},$$

where *m* and *t* are the number of muscles and the number of sampling data, respectively. The number of commands for controlling *m* muscles can be reduced by using the following matrix of the muscle synergy *W*:

(2) M=WC+E,

Where

(3) $$W\in R^{m\;\times \;n},\;C\in R^{n\;\times \;t},\;E\in R^{m\;\times \;t}$$

and *n* is the number of control commands.

We assume that *W* is normalized to satisfy the following conditions:

(4) $$\begin{array}{l} \;W=\left[W^{\left(1\right)}W^{\left(2\right)}W^{\left(3\right)}\cdots W^{\left(n\right)}\right],\\ |W^{\left(i\right)}|\;=\;1, \end{array}$$

where *W*^(*i*)^ denote the vector expressed as

(5) $$W^{\left(i\right)}\in R^{m}.$$

**Figure 3:**
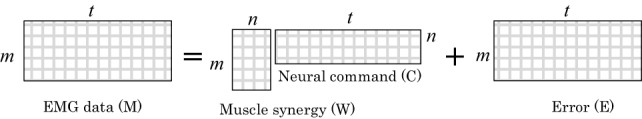
**A conceptual-mathematical model for identifying muscle synergies**.

We set *n* to be smaller than *m*. Equation 1 implies that *n* commands are used to control *m* muscles by using the muscle synergy *W* (Figure 3).

*C* is the matrix containing the *n* commands to control *m* muscles as follows:

(6) $$\begin{array}{l} C=\left[\begin{array}{c} C^{\left(1\right)}\\ C^{\left(2\right)}\\ C^{\left(3\right)}\\ \vdots \\ C^{\left(n\right)} \end{array}\right],\\ \;C^{\left(i\right)}\in R^{t} \end{array}$$

The error between *M* and *WC* is expressed as ***E***, which must be small enough that *m* muscles are controlled by *n* commands in Equation 1. The magnitude of ***E*** is described by an index of *similarity L*, which is sensitive to both the shape and magnitude of the measured and reconstructed muscle patterns (Torres-Oviedo and Ting, 2007):

(7) $$L=100\left(1-\frac{1}{m}\sum_{i=1}^{m}\frac{\sqrt{\frac{1}{t}\sum_{j=1}^{t}E_{ij}^{2}}}{\sqrt{\frac{1}{t}\sum_{j=1}^{t}M_{ij}^{\prime^{2}}}}\right),$$

where

$$M^{\prime }=WC,$$

and *E*_*ij*_ and *M*′_*ij*_ are the elements of matrices *E* and *M*′,respectively. The range of *L* is 0 < *L* < 100. When the magnitude of ***E*** becomes smaller, *L* becomes larger. We considered a value of *L* > 75% to indicate a good fit with the original data (Torres-Oviedo et al., 2006). This criterion ensured that each muscle would be well reconstructed. A reasonable value of *n* is chosen by using the index *L* through the non-negative matrix factorization algorithm (Lee and Seung, 2001).

*W*^(*i*)^ in Equation (2) is regarded as the base vectors of the space created by *W*. We call this space a *synergy space*. *C* is the commands moving in the synergy space. We assume that *m* muscles are controlled in this *n*-dimensional space to simplify their controls.

### Indices for analyzing muscle synergy

To analyze APR behavior based on muscle synergy, we use two indices: *SSI* and *SCI*. *SSI* indicates the similarity in muscle synergy between multiple *APRs*, and *SCI* indicates the size of the synergy space.

#### Synergy stability index

*SSI* is describes the similarity in muscle synergy between the several APRs conducted by a same participant. *SSI* is calculated by using Pearson's correlation coefficient:

(8) $$SSI=\frac{1}{n}\sum_{i\;=\;1}^{n}\left(\frac{2}{p(p-1)}\sum_{l\;\neq \;q}^{p}r\left(W_{l}^{\left(i\right)},\;W_{q}^{\left(i\right)}\right)\right).$$

Here, *W*_*j*_ is the muscle synergy of the *j*th behavior (*j* = 1 … *p*), and the Pearson's correlation coefficient is defined as:

(9) $$r(x,\;y)=\frac{\sum_{i\;=\;1}^{m}\left(x_{i}-\bar{x}\right)\left(y_{i}-\bar{y}\right)}{mS_{x}S_{y}},$$

where *x* and *y* are two vectors to be compared, *x* and *y* are their mean values, and *S*_*x*_ and *S*_*y*_ are their standard deviations. *SSI* has the following range:

0≤SSI≤1.

A high *SSI* value indicates that the participant uses similar muscle synergies in all motions. In the case where the synergies of all motions are completely the same, *SSI* equals to 1. To avoid the ordering issue of the resulting synergies, we were re-sorting the resulting synergies so to produce the highest *SSI*.

*SSI* is also applicable to describing the stability of neural command ***C*** in Equation 1. The following equation is used to compute the stability of ***C***:

(10) $$SSI_{c}=\frac{1}{n}\sum_{i=1}^{n}\left(\frac{2}{p(p-1)}\sum_{l\neq q}^{p}r\left(C_{l}^{\left(i\right)},\;C_{q}^{\left(i\right)}\right)\right),$$

where ***C***_*j*_ is the neural command of *j*th motion. The range of *SSI*_*C*_ is from 0 to 1. Where the 1 means that the C's are the same on multiple movements.

#### Synergy coordination index

*SCI* is used to evaluate the size of the resulting synergy space, or in other words, the coordination between the utilized synergies. Let us assume that muscle synergy *W* is expressed as

$$W=\left[W^{\left(1\right)}\;W^{\left(2\right)}\;W^{\left(3\right)}\;\cdots \;W^{\left(n\right)}\right],$$

where *W*^(*i*)^ ∈ *R*^*m*^ is the base vector of the synergy space. Because we use the non-negative matrix factorization algorithm to estimate *W*, the synergy space exists for only positive vector components. Furthermore, vectors *W*^(*i*)^(*i* = 1 … *n*) are in general not orthogonal to each other. The size of the synergy space depends on the relative angles of the vectors *W*_*i*_. To quantify the size of the synergy space, we define *SCI* by using the inner product of *W*_*i*_:

(11) $$SCI=\frac{2}{n(n-1)}\sum_{i\neq j}^{n}W^{\left(i\right)}W^{\left(j\right)}.$$

The range of *SCI* is 0 ≤ *SCI* ≤ 1. *SCI* = 1, implies that all vecto*rs W*^(*i*)^ are identical, whereas *SCI* = 0, implies that all vectors *W*^(*i*)^ are orthogonal each other. Therefore, as shown in Figure 1C the synergy space becomes smaller when *SCI* becomes larger.

## Results

### Dimensions of synergy space

All subjects successfully completed the assigned tasks. Our next step was to identify the appropriate dimensions of the synergy space of each participant, or in other words, the number of synergies that represent the recorded EMG patterns. To do so, first we used the recorded EMG data to calculate all possible synergies of each participant in each successful trial, and second we reconstructed back muscle activations based on those computed synergies.

**Figure 4:**
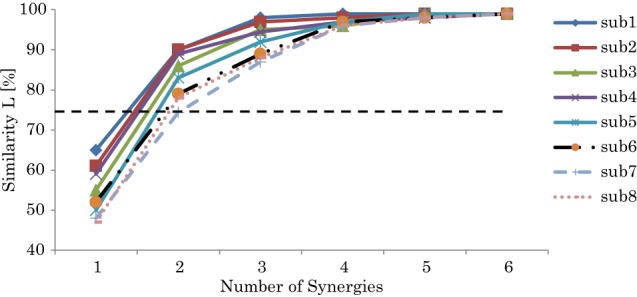
**Similarity L between the recorded and reconstructed muscle activation patterns from all possible computed synergies**. The plots show the means and SD across participants. The dashed line indicates the threshold *L* > 75%.

Based on the quantitation of Equation 7, Figure 4 shows the similarity *L* between the recorded and reconstructed muscle activations from the possible computed synergies of participants. The minimum number of synergy space dimensions giving similarity in excess of the threshold value (*L* > 75%) was two synergies for all the subjects (we have also tested different thresholds to validate our threshold selection; see the supplementary materials for details). Accordingly, we assumed that the collected muscle patterns from the participants could be enough represented by two-dimensional synergy spaces. From the figure, we can note the correlation between the decomposition errors of the subjects and their balance skill, see the scores in Figure 7.

**Figure 5:**
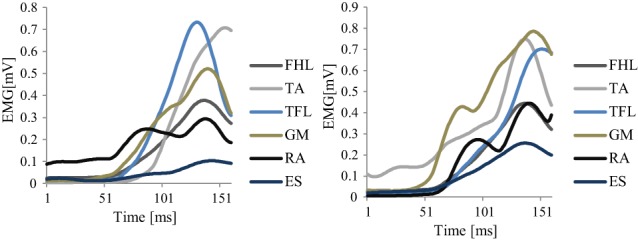
**The recorded EMG from 2 different trials of a representative good performer (sub.2)**.

**Figure 6:**
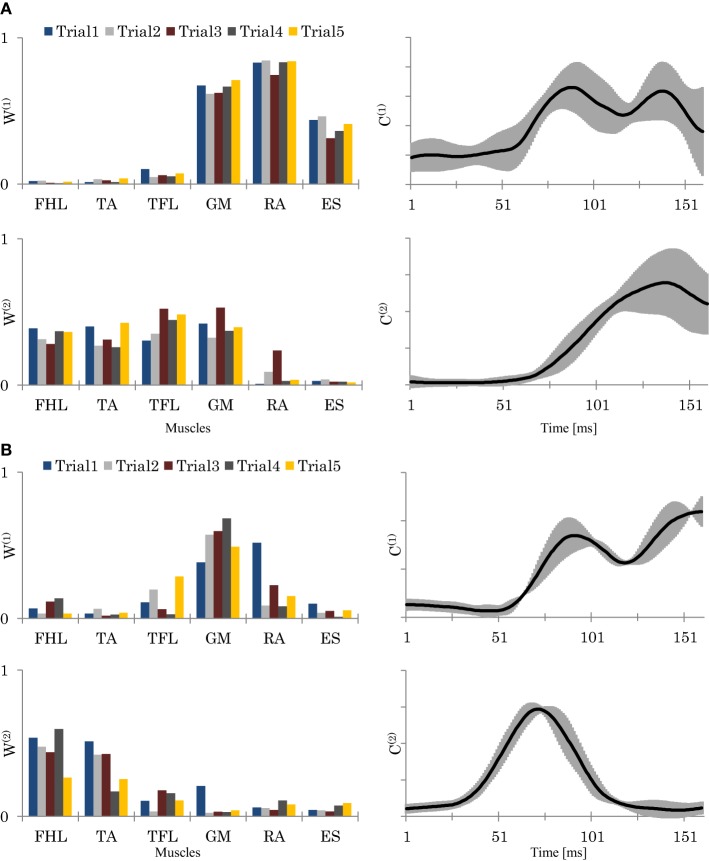
**The resulting synergies for two representative subjects (Sub.2, *L* = 89%) (A), and (Sub.8, *L* = 78%) (B)**. Left column: the resulting synergy space *W* from 5 trials. Right column: the resulting neural command *C* (means and SD of the 5 trials). In **(A)**, Sub.2, seemed to utilize a hip strategy by *W*^(1)^, and ankle and hip strategies by *W*^(2)^. In contrast, in **(B)**, sub.8, seemed to utilized only the ankle strategy by *W*^(2)^. *C*^(1)^ and *C*^(2)^, represents each synergy utilizing time.

Figure 5 shows an example of the original EMG data of two different trials recorded from a representative good performer. Figure 6 shows an example of the resulting synergies of two representative subjects; good performer and bad performer. From Figures 5 and 6, we can observe that the recorded original EMG data is variable on all subjects regardless the quality of their responses.

### Synergy characteristics

**Figure 7:**
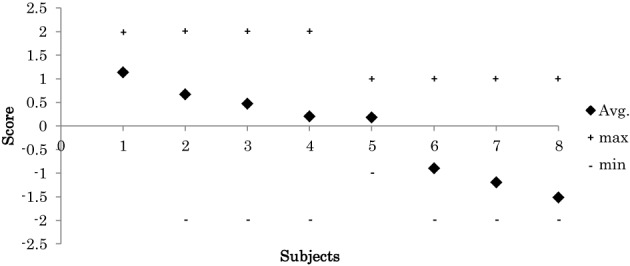
**Participant's score (mean, maximum “+” and the minimum “−” scores across all trial)**.

During experiments, the 8 participants exhibited various skill levels in response to the displacement of the platform. In each trial, we visually evaluated each participant's response according to the criteria in Table 1. Figure 7 shows the mean of each participants scores across the successful trials. The results show that the participants had various levels of balance skill.

**Figure 8:**
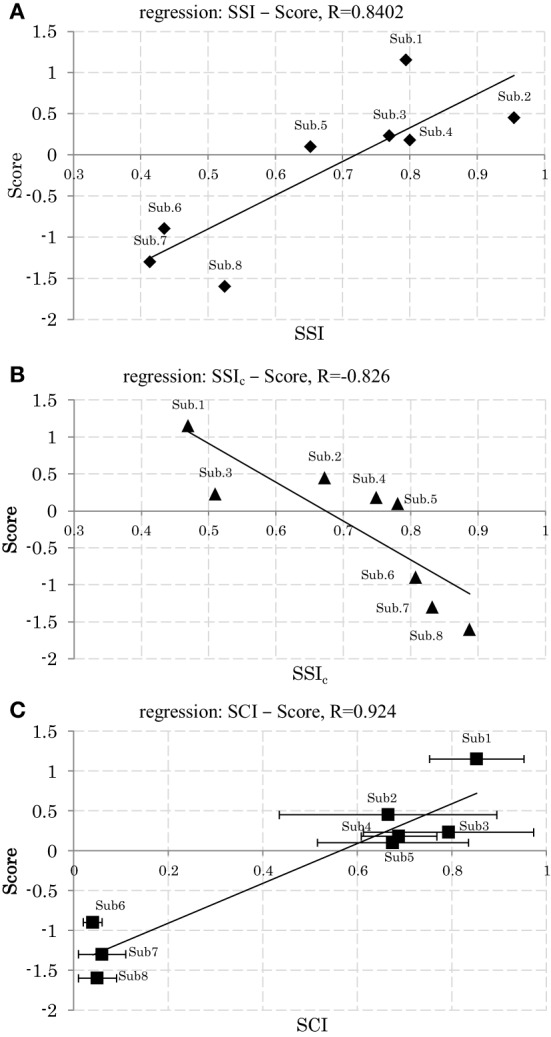
**(A)** Relation between synergy stability index (*SSI*) and the scores. **(B)** Relation between synergy stability index of the neural command (*SSI*_*c*_) and the scores. **(C)** Relation between synergy coordination index (*SCI*) and the scores (Mean and SD across 5 trials.). Linear least-squares regression line.

Figures 8A,B shows the relationships of *SSI* and *SSI*_*c*_ with the participants' scores. Here we see that participants with a high score (low score) showed lower (higher) *C* stabilities and higher (lower) *W* stabilities, respectively. Figure 8C shows also the relationships of *SCI* with the participants' scores. The plots here reveal that participants with a higher score used a smaller size of synergy space to respond to the disturbance, whereas those with a lower score used a larger size. Figures 6A,B show an example of the resulting synergies of higher and lower scores participants, respectively. In the higher score participant, the resulting synergy space in each trial are significantly similar (*SSI* = 0.95). On the other hand, the stability of the recruiting neural commands are lower (*SSI*_*c*_ = 0.67). The lower score participant, however, showed the opposite, Figure 6B.

From these results we may conclude that stability level of *C* and *W* could be a possible index to reflect participants' skills to maintain balance against the sudden disturbance. Regardless the variability of original EMG data on all subjects, high scores participants used almost fixed region of the synergy space across trials combined with a variable representation of the neural command *C*, while low scores participants utilized variable regions of the synergy spaces combined with a fixed representation of the neural command *C*. Furthermore, high scores participants used smaller size of synergy space than low scores participants.

### Training experiment

In the second experiment, we aimed to observe the changes of muscle synergy during training. We asked the participants with the lowest scores (i.e., participants 6, 7, and 8) to continue performing a similar set of experimental trials for additional five sessions. Each session was conducted every two days. At each session, we requested the participants to do additional pre- and post-session practice on the platform for roughly 80 trials interspersed with adequate rest periods. Electrode positions on the participant's body were marked at each session to ensure similar electrode placement in the next experimental session.

**Figure 9:**
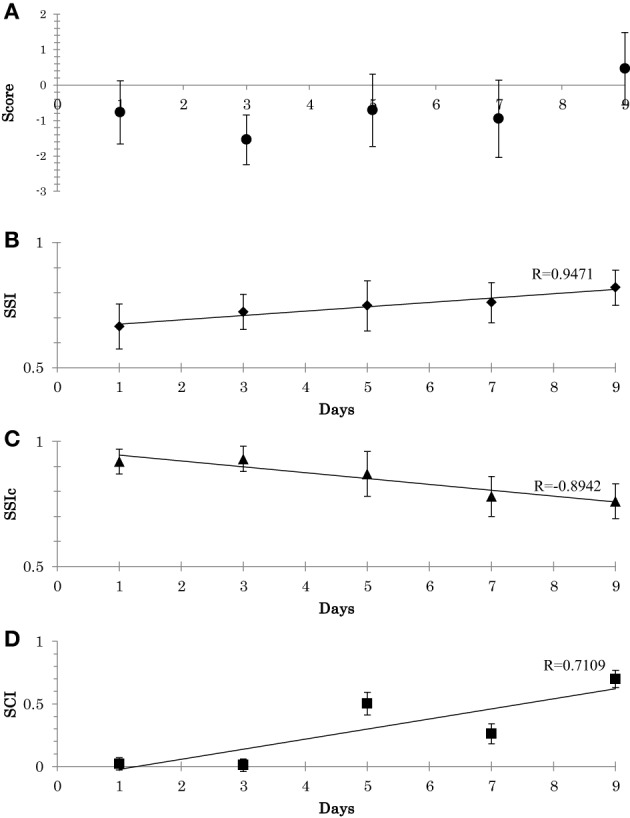
**(A)** Participant's scores during the adaptation experiment (mean and the SD across all trial). **(B)** Adaptation of synergy stability index (SSI) along the 9 days (Mean and SD across the 3 subjects). **(C)** Adaptation of synergy stability index of the neural command (SSIc). **(D)** Adaptation of synergy coordination index (SCI). Linear least-squares regression line.

Experimental results were as follows. Firstly, regarding the balance scores, participants show improvements in their scores, Figure 9A. Secondly, with respect to *SSI* and *SSI*_*c*_, a gradual decrease in the stability of *C* and a gradual increase in the stability of *W* occurred, see Figures 9B,C. Thirdly, with respect to *SCI*, gradual decrease was observed in the synergy size (i.e., an increase in *SCI*), Figure 9D. From the Figure 9, the scores, *SSI*, and *SCI* were associated with one another; thus, the overall relationships were fairly consistent with the outcomes presented in the above experiment.

From this training experiment, we can highlight two main points: (1) synergies seem to be altered through systematic training: the adaptation of *W* occurs until a proper region is located. This stage occur in conjunction with the adaptation of *C*. (2) The CNS appears to force the recruitment direction of the muscle synergy to ensure higher coordination between the utilized synergies (i.e., a smaller size of synergy space), which could be the reason for the resulting quality of the subsequent behaviors (Video1, appendix: shows the behavior of a representative subject before and after the training).

## Discussion

Results show various *SSI* for both synergy space (*W*) and neural commands (C), which were both associated with participant balance skill. Participants with a high score exhibited higher stability of *W* (high *SSI*) and lower stability of *C* (low *SSI*_*c*_), and vice versa. These findings were also reiterated in a training experiment: participants with an initially low score could improve their score by creating the new synergy spaces through training.

The value of *SCI* also appears to encode essential factors regarding the quality of the behavior. Participants with high scores exhibited remarkably smaller size of synergy spaces, and vice versa for the participants with low scores.

To ensure that these designed indices reflect universal features about the muscle synergy and is not just the artifact of EMG data decomposition, we applied the indices on various dimensions of synergy spaces (see the supplementary materials for details). Results showed that *SSI* increased as the dimension of the synergy space increased by increasing the threshold. The increase of the dimension implies that the variability of the data comes closer to the variability of the original EMG data. *SCI* also increased as the dimension of the synergy space increased. In *SCI*, we assume that the same data is decomposed in the different number of vectors. The average of the spaces between the vectors in *W* gets smaller when the number of vectors is increased. The analyses of *SCI*, however, show us another possibility of *SCI*. *SCI* can represent how much the current dimension is close to the lower or higher dimensional space. Thus, it might be used as the sub-scale of the dimension of muscle synergy.

Our results suggest that the behaviors of the participants with good balance skills were recruited from the appropriate region and size of synergy space, which were indicated by *SSI* and *SCI*, respectively. The improvements of *SSI* and *SCI* through the balance training show that the appropriate region and size of the synergy space are learnable. We think that the learning of synergy space region and size by the training depend on individuals because of the difference of the body parameters such as the body heights, weights and the strength of muscles.

### Learning model

These resulting characteristics of *SSI*, SSI_*c*_, and *SCI* reveal an important learning methodology of muscles, which we summarized in the following four points:

First, when participants were introduced to the task; if the proper region and size of their synergy space for responding to the task were not yet solved (e.g., in participants with low scores), the CNS tried at each trial to search for the most appropriate synergy space region, evoking various strategies by tuning the value of *W*. Due to this search stage, low stability of *W* was observed.

Second, the search criterion that the CNS appeared to rely on in this stage was the size of the synergy space. Narrow space appeared to be the desired target of the CNS. Thus, high *SCI* was observed in the participants with higher scores. After a number of search trials throughout training, the appropriate coordination of the muscles gradually emerged, and a gradual increase in the stability of *W*.

Third, during the search stage of *W*, and to simplify handling of its high temporal variability, the CNS attempted to reduce the degrees of freedom of the resulting motions by restricting the variability of *C*, and thus, high stability of *C* was observed. One possible mechanism that might be used by the CNS to manage this stage could be decreasing sensitivity to changes caused by sensory inputs. Such sensitivity reduction can necessitate extra energy from CNS to maintain high stability of *C* (considering that the inputs were variable in nature).

Fourth, eventually after learning, *W* was stable in a particular region and smaller in size, and the constraint on the variability of *C* was gradually eased. Thus, a reduction in the stability of *C* occurred as a natural response to the variability from the input side.

Our findings could be consistent to some extent with the individual learning stages reported by Bernstein (1967), where individuals learn motor coordination first by temporarily restricting the degrees of freedom that they use. This enables the learner to simplify the dynamics of the involved body parts and the range of movement options when searching for the optimal muscle combinations. Once the individual has gained a certain level of proficiency, the constraint can be relaxed, thereby allowing them to use the full potential of their body (decreased variability of *W* and increased variability of *C*).

The resulting variability of *W* and *C* in this study could be also associated to the bad and good variability hypothesis discussed by Latash and Anson (2006). This hypothesis mainly indicates that variability is naturally presented in human movements. Thus, bad variability is the one which affects the final performance results of the motor task. Good variability, on the other hand, always works to achieve better outcome. The CNS, therefore, may, in our case, works to decrease the bad variability *W* and increase the good variability *C*.

## Future directions

Although the above results offer hints to the possible learning method of muscle synergies, an open question remains: what is the criterion that the CNS uses to recruit muscle synergies taking into account environment interaction inputs? To answer this question, we are currently working to expand the concept of muscle synergy, to include the concept of *sensory synergy*, the form of input to the CNS (Ting, 2007; Alnajjar et al., 2012). The idea behind this extension is that, if the CNS deals with body muscles through muscle synergies, the possibility should be explored as to whether the CNS deals with body sensory receptors through sensory synergies as well.

Recently, several studies have shown that the activation of muscle synergies correlates with environment inputs (Burgess et al., 1982; Ivanenko et al., 2003; Krishnamoorthy et al., 2003). Krishnamoorthy et al., for instance, examined a possible correlation between muscle synergies and center of pressure displacement. Ivanenko et al. also examined the correlation between muscle synergies and endpoint foot kinematics during locomotion. In these studies, the concept of input synergies has not yet been fully discussed, and therefore, we are intending to addressed it in our future direction and investigate its relationship with our current results.

With the aim of developing an effective neurorehabilitation model, we believe that our current results, as a preliminary learning model, will lay the groundwork for a new avenue of research toward understanding the CNS and motor learning. Linking these results to classify post-stroke patients based on their impairment level and the positional of recovery, as well as, building rehabilitation-training robots to assist them is a goal for future work.

## Authors contribution

Conceived and designed the experiments: Fady Alnajjar, Tytus Wojtara, Hidenori Kimura, and Shingo Shimoda. Performed the experiments: Fady Alnajjar, Tytus Wojtara. Analyzed the data: Fady Alnajjar, Tytus Wojtara, and Shingo Shimod. Formed the Equations: Fady Alnajjar, and Shingo Shimod. Wrote the paper: Fady Alnajjar. Revised the paper: Fady Alnajjar, and Shingo Shimod.

### Conflict of interest statement

The authors declare that the research was conducted in the absence of any commercial or financial relationships that could be construed as a potential conflict of interest.
