# Supplementary material for: Muscle synergy space: learning model to create an optimal muscle synergy
Source: Front Comput Neurosci. 2013 Oct 15;7:136. doi: 10.3389/fncom.2013.00136 (PMC3796759; doi:10.3389/fncom.2013.00136)
Supplement: Supplementary file 2 [file Presentation1.PDF]

TITLE: MUSCLE SYNERGY SPACE: LEARNING MODEL TO CREATE AN OPTIMAL MUSCLE SYNERGY

AUTHORS: Fady Alnajjar, Tytus Wojtara, Shingo Shimoda, Hidenori Kimura

SUPPLEMENTARY MATERIALS

Selection of the threshold value

To validate the universal features of the designed indices, here we compare between L=75%, 80%, or 90%. Fig.1 shows the value of L from all the computed synergies of the 8th subjects (means across the recorded trials). From the figure, at the threshold of 75%, all subjects use the 2 synergies. At the threshold of 80%, good performers shows 2 synergies (Sub1~Sub5), and bad performers shows 3 synergies (Sub6~Sub8). Finally, threshold of 90%, good performers shows 2/3 synergies (Sub1: 2 Synergies, Sub2~Sub5: 3 Synergies), and bad performers shows 4 synergies (Sub6~Sub8).

Fig.2 shows the changes in both SSI and SSIc at the threshold of L= 90% compared to L=75%. From the figure, when the threshold increased, the dimensions of the synergy space increase. The increase of the dimension implies that the variability of the data comes closer to the variability of the original EMG data. Thus, SSI got smaller when the threshold was changed to the higher value.

Fig.3 shows the SCI of the utilized synergies in L=75%, 80%, or 90%. From the figure, the value of SCI is increased as the number of utilized synergies to compute it is increased.

From these results we can conclude that our designed indices can encode important features to characterize the resulting synergies on the basis of the quality of the resulting behavior.

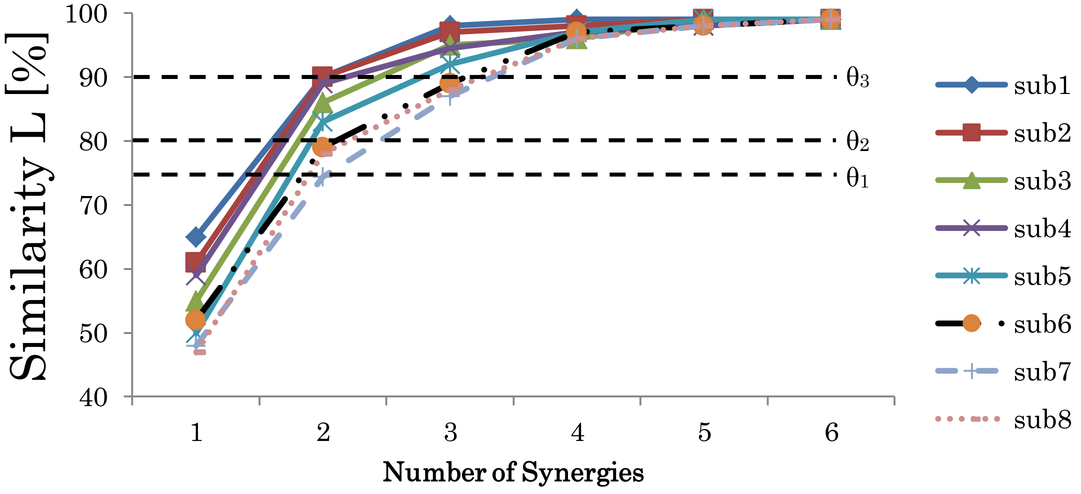

FIG.1. Similarity *L* between the recorded and reconstructed muscle activation patterns from all possible computed synergies (the plots show the means across recorded trials).

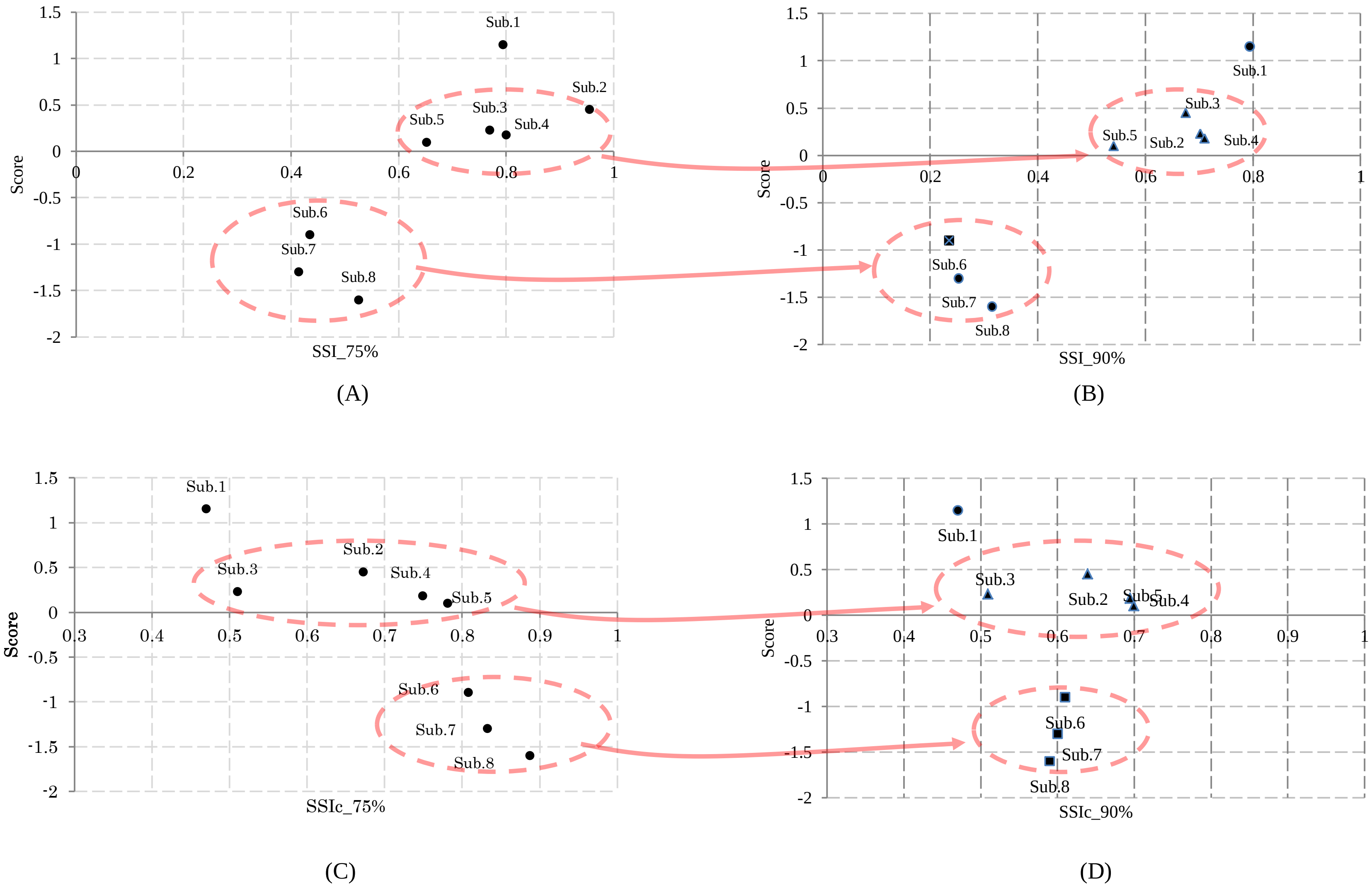

FIG.2. SSI, SSI_C and the scores (Mean). The circle, triangle, and square plots indicate the index which were calculated from two, three, or four synergies, respectively.

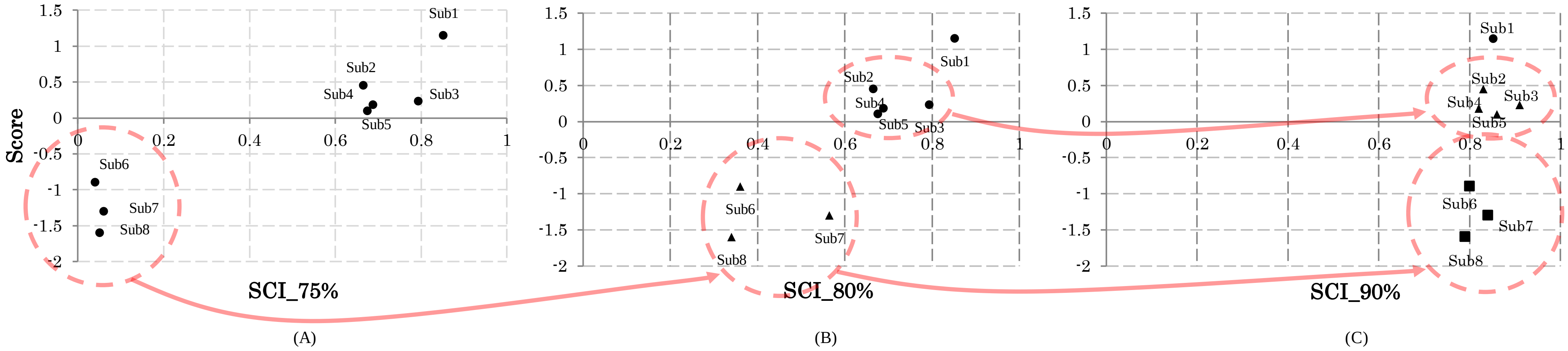

FIG.3. SCI and the scores (Mean). The circle, triangle, and square plots indicate the SCIs which were calculated from two, three, or four synergies, respectively.
